# Programmed death-ligand 1 (PD-L1) polymorphisms as predictive biomarkers for the development of liver cirrhosis and hepatocellular carcinoma in HCV Egyptian patients

**DOI:** 10.1016/j.tvr.2022.200249

**Published:** 2022-10-18

**Authors:** Marwa Hassan, Mohammed Saad Attia, Zainab Ali-Eldin, Gamal El Attar, Mohamed Elzallat, Hany Haroun Kaisar Saad, Amira Isaac

**Affiliations:** a Immunology Department, Theodor Bilharz Research Institute, Giza, Egypt; b Hepato-Gastroenterology Department, Theodor Bilharz Research Institute, Egypt; c Internal Medicine and Hepato-Gastroenterology Department, Faculty of Medicine, Ain Shams University, Egypt

**Keywords:** Gene polymorphism, Hepatitis C virus, Hepatocellular carcinoma, Liver cirrhosis, Programmed death-ligand 1

## Abstract

**Background:**

Considering the immune evasion role of programmed death-ligand 1 (PD-L1) in cancer development, its genomic variations might be closely associated with disease development and cancer risks. Accordingly, this study was performed to investigate how the PD-L1 gene polymorphisms affect the susceptibility to hepatitis C virus (HCV)-induced liver cirrhosis and cancer development in the Egyptian population.

**Methodology:**

Two single nucleotide polymorphisms of the PD-L1 gene; rs2297136 (A > G) and rs4143815 (C > G), were studied in 50 HCV, 51 liver cirrhosis, and 52 hepatocellular carcinoma (HCC) patients as well as 50 healthy subjects using real-time PCR.

**Results:**

The frequencies of PD-L1 rs2297136 AA and rs4143815 GG genotypes were significantly higher in the liver cirrhosis than the control and HCV groups. The rs4143815 CG and GG genotypes were linked to a higher risk of developing HCC and were positively associated with the clinicopathological features of HCC.

**Conclusions:**

The PD-L1 rs2297136 AA and rs4143815 GG genotypes increase the susceptibility to liver cirrhosis. The rs4143815 CG and GG genotypes are positively associated with HCC risk and its clinicopathological characteristics. Therefore, HCV patients carrying the PD-L1 rs4143815 G-allele should be followed up on a regular basis to allow for early HCC management.

## 1 Introduction

Hepatocellular carcinoma (HCC) is a chief cause of cancer-associated morbidity and mortality and represents a major health concern worldwide. According to GLOBOCAN 2020, HCC is ranked as the 7th most incident neoplasm and the 3rd cause of cancer-related death [1]. In a significant proportion of cases, it is diagnosed at delayed stages and more than half of patients with a localized disease will develop disease recurrence after loco-regional therapeutic modalities [2].

Chronic inflammation in the liver appears to establish an immunosuppressive milieu that promotes the HCC tumorigenesis and progression, suggesting that targeting the dysregulated tumor microenvironment could improve the response to HCC treatment [3]. Many studies are currently being conducted to examine the mechanisms of HCC immune evasion via the programmed death receptor 1 (PD-1)/programmed death-ligand 1 (PD-L1) pathway.

PD-1 is an immunosuppressive receptor found on activated T cells, B cells, NK cells, T regulatory cells (Tregs), myeloid-derived suppressor cells (MDSCs), and dendritic cells. It was originally assumed to be a receptor that causes activated T cell death, hence the term programmed cell death protein [4]. However, it was eventually discovered that it is an immunological checkpoint with a negative regulatory role. It has two ligands: The first one is PD-L1 (CD274 or B7–H1) which is generally expressed on a variety of somatic and immunological cells, including antigen-presenting cells (APCs) and endothelial cells. It is primarily responsible for suppressing T-cell migration, proliferation, and cytotoxic mediator secretion. The second ligand of the PD-1 receptor; PD-L2 (CD273 or B7-DC), is infrequently expressed on APCs [5,6].

As an adaptive mechanism to evade the host immune surveillance, cancer cells have evolved to hijack the PD-1/PD-L1 signaling by constitutively expressing PD-L1 or PD-L2 to activate the inhibitory receptor PD-1 found on the tumor-infiltrating lymphocytes (TILs). In patients who have never received immunotherapy, overexpression of PD-L1 on hepatic malignant cells has been linked to tumor recurrence, aggressiveness, vascular invasion, and a poor prognosis [7]. The PD-1/PD-L1 ligation inhibits immune cell activation as well as the generation of specific inflammatory cytokines such as IFN-γ, resulting in immunological suppression and peripheral tolerance. Immunotherapies that target the PD-1/PD-L1 pathway have demonstrated promise success in a variety of cancers, including HCC [8].

The PD-L1 gene is polymorphic and many studies have investigated the association between the genetic variants of the PD-L1 gene and the susceptibility to different types of cancer in distinct ethnic populations, but the findings are still equivocal [9,10]. As a result, this study was designed to detect the relationship between the genetic variations of PD-L1 (rs4143815 and rs2297136) and the risk of developing liver cirrhosis and HCC in the Egyptian population suffering from chronic hepatitis C virus (HCV) illness.

## 2 Materials and methods

### 2.1 Subjects of the study

A total of 153 patients including 50 HCV-infected patients, 51 HCV-associated liver cirrhosis patients, and 52 HCV-associated HCC patients, recruited from theHepato-Gastroenterology Department, Theodor Bilharz Research Institute, were enrolled in this study, in addition to 50 age- and sex-matched healthy controls. Written informed consents were obtained from all subjects as stated by TBRI's Human Research Ethics Committee and in compliance with the guidelines of the 1975 Declaration of Helsinki. The following criteria were met by all of the patients: (a) over the age of 18 and (b) proven to be HCV genotype 4 positive. They were assigned to the HCV group if they didn't have any radiological evidence of liver cirrhosis or hepatic lesions, to the HCV-associated liver cirrhosis group if they had radiological evidence of liver cirrhosis, and to the HCV-associated HCC group if they had a malignant hepatic lesion that was detected by ultrasonography and confirmed by a triphasic abdominal CT. The healthy controls were employees free from hepatitis viral infections and liver disorders.

If the participants had clinical or laboratory evidence of other etiologies of chronic liver disease, such as Schistosoma infection or HBV infection, or other malignancies, they were considered ineligible for this study.

All subjects had a detailed medical history, clinical examination, routine laboratory tests, serological diagnosis of Schistosomiasis and viral hepatitis, α-fetoprotein measurement, HCV-RNA RT-PCR, ultrasonography, and triphasic abdominal CT for HCC confirmation. All patients were scored using: (a) Child-Pugh score based on serum albumin, bilirubin, prothrombin time, degree of ascites, and encephalopathy; (b) Model for End-Stage Liver Disease/Na (MELD/Na) score based on serum bilirubin, creatinine, INR, and sodium; and (c) Barcelona Clinic Liver Cancer (BCLC) staging based on the number and size of hepatic focal lesions and portal vein invasion.

### 2.2 DNA extraction and SNP genotyping

Blood samples were obtained from all subjects, and genomic DNA was isolated with the QIAmp DNA Mini Kit (Qiagen, Hilden, Germany) according to the manufacturer's protocol. The extracted DNA was eluted in TE buffer and stored at - 80 °C for further use. Two SNPs of PD-L1 gene, including rs2297136 (A > G) and rs4143815 (C > G), were examined by the real-time polymerase chain reaction (real-time PCR). The SNPs selection was based on those previously evaluated in relation to cancer or those with evidence of functional significance. The PCR mixture was prepared for each sample by using 10 μL TaqMan™ Universal Master Mix II (Thermo Fisher Scientific, USA), 0.5 μL of TaqMan™ SNP Genotyping Assay (40X) (ready-made assay, Cat. No: 4351379, Thermo Fisher Scientific, USA), 1 μL genomic DNA, and 8.5 μL RNase-free water to reach a total volume of 20 μL. A negative control consisting of 10 μL TaqMan™ Universal Master Mix II, 0.5 μL of TaqMan™ SNP Genotyping Assay (40X), and 9.5 μL RNase-free water was also prepared for each SNP. The real-time PCR cycling was initiated with UNG incubation at 60 °C for 2 min, polymerase activation at 95 °C for 10 min, followed by 40 cycles of (denaturation at 95 °C for 15 s and annealing at 60 °C for 1 min).

### 2.3 Statistical analysis

The statistical analyses were carried out with the statistical SPSS Package program version 25 for Windows (SPSS Inc., Chicago, IL). The Hardy-Weinberg test was used to determine the heredity equilibrium. The quantitative descriptive statistics, including the mean and standard error (SE), were done for all the quantitative variables while the qualitative descriptive statistics, such as the frequency and percentage, were performed for all the qualitative variables. For the quantitative variables, the ANOVA test, followed by Tukey HSD, was done to compare the different groups. All the qualitative characteristics, including genotype distributions, were compared between the four groups using the Chi-square test. The odds ratio (OR) and a 95% confidence interval were used to calculate the genotype relative risk. At the 0.05 level of probability (*p* < 0.05), all the statistical analyses were considered significant.

## 3 Results

This study incorporated 203 participants divided into four groups; HCV Group (n = 50), HCV-associated liver cirrhosis group (n = 51), HCV-associated HCC group (n = 52), and age- and sex-matched normal control group (n = 50), with 118 males and 85 females aged between 42 and 84 years old. The demographic characteristics of the four groups revealed that there was no statistically significant difference in age, gender composition, smoking, and diabetes across the groups tested. Table 1 shows the demographic, laboratory, and radiological data for the groups analyzed.

**Table 1:** The demographic, laboratory, and radiological data of the studied groups.

Variables	**Control**	**HCV**	**HCV + Cirrhosis**	**HCV + HCC**
	**(n = 50)**	**(n = 50)**	**(n = 51)**	**(n = 52)**
**Age (year)**	60.28 ± 1.15	59.86 ± 1.01	61.08 ± 1.12	63.48 ± 1.08
**Gender**				
Males	28 (56.0%)	27 (54.0%)	25 (49.0%)	38 (73.1%)
Females	22 (44.0%)	23 (46.0%)	26 (51.0%)	14 (26.9%)
**Smoking**				
No	32 (64.0%)	30 (60%)	38 (74.5%)	29 (55.8%)
Yes	18 (36.0%)	20 (40%)	13 (25.5%)	23 (44.2%)
**Diabetesmellitus**				
No	37 (74.0%)	34 (68.0%)	30 (58.8%)	33 (63.5%)
Yes	13 (26.0%)	16 (32.0%)	21 (41.2%)	19 (36.5%)
**Routinelaboratory tests**				
AST (IU/L)	23.32 ± 0.99	54.72 ± 3.03	61.00 ± 6.51	191.98 ± 45.72**^,^ *
ALT (IU/L)	24.06 ± 1.38	41.66 ± 2.20	34.78 ± 5.32	84.85 ± 13.69^@^
Total bilirubin (mg/dl)	0.62 ± 0.04	2.03 ± 0.19	2.86 ± 0.56	5.50 ± 1.05**^, $^
Serum albumin (g/dl)	3.99 ± 0.05	3.53 ± 0.09^a^	2.49 ± 0.08^a, b^	2.51 ± 0.08^a, b^
Total protein (g/dl)	7.33 ± 0.08	6.63 ± 0.09^a^	6.55 ± 0.12^a^	6.93 ± 0.14^c^
PC (%)	93.12 ± 0.76	79.32 ± 2.83^a^	55.83 ± 2.43^a, b^	55.33 ± 2.69^a, b^
AFP (ng/ml)	4.45 ± 0.25	5.43 ± 0.47	10.79 ± 1.70	15219.75 ± 3888.43^@^
**Scoring of patients**				
**Child-Pugh score**				
A	–	–	7 (13.7%)	6 (11.5%)
B	–	–	14 (27.5%)	12 (23.1%)
C	–	–	30 (58.8%)	34 (65.41%)
**MELD/Na score**				
≤ 9	–	–	5 (9.8%)	5 (9.6%)
10–19	–	–	28 (54.9%)	18 (34.6%)
20–29	–	–	13 (25.5%)	18 (34.6%)
≥ 30	–	–	5 (9.8%)	11 (21.2%)
**BCLC staging**				
A	–	–	–	2 (3.8%)
B	–	–	–	7 (13.5%)
C	–	–	–	11 (21.2%)
D	–	–	–	32 (61.5%)
**Lesion size**				
< 3 cm	–	–	–	13 (25.0%)
≥ 3 cm	–	–	–	39 (75.0%)

Quantitative data are expressed as mean ± standard error (SE).

Qualitative data are expressed as frequencies (percentages).

Hepatocellular carcinoma (HCC); aspartate aminotransferase (AST); alanine aminotransferase (ALT); prothrombin concentration (PC); alpha-fetoprotein (AFP).

**Significant increase compared to the control and HCV groups (*p* < 0.001); *Significant increase compared to the cirrhosis group (*p* < 0.01); ^@^Significant increase compared to the control, HCV, and cirrhosis groups (*p* < 0.001); ^$^Significant increase compared to the cirrhosis group (*p* < 0.05); ^a^Significant decrease compared to the control group (*p* < 0.001); ^b^Significant decrease compared to the HCV group (*p* < 0.001); ^c^Significant decrease compared to the control group (*p* < 0.05).

The genotype distributions of the two PD-L1 gene polymorphisms, rs2297136 (A > G) and rs4143815 (C > G), in the studied groups, are summarized in Table 2, Table 3. Based on the Hardy-Weinberg equilibrium, the genotype distributions of the tested SNPs were in a balanced state in all groups (*p* > 0.05). The rs2297136 (A > G) SNP AA genotype was associated with a significantly elevated risk of liver cirrhosis compared to the control and HCV groups (OR = 2.96, 95% CI = 1.28–6.84, *p* = 0.01 and OR = 2.67, 95% CI = 1.17–6.011, *p* = 0.018, respectively). However, no association was detected between the rs2297136 (A > G) SNP and HCC development (Table 2).

**Table 2:** The genotypes distribution of rs2297136 (A > G) polymorphism in the studied groups.

	**Control**	**HCV**	**HCV + Cirrhosis**	**HCV + HCC**	***P*****value**
	**(n = 50)**	**(n = 50)**	**(n = 51)**	**(n = 52)**	
**Genotype**					
**AG**	26 (52%)	28 (56.0%)	16 (31.4%)	24 (46.2%)	0.078
**AA**	13 (26%)	14 (28.0%)	26 (51%)	22 (42.3%)	
**GG**	11 (22%)	8 (16.0%)	9 (17.6%)	6 (11.5%)	
**Genotype**					
**AG**	26 (52.0%)	28 (56.0%)	16 (31.4%)	24 (46.2%)	0.068
**AA + GG**	24 (48.0%)	22 (44.0%)	35 (68.6%)	28 (53.8%)	
**Genotype**					
**AA**	13 (26%)	14 (28%)	26 (51%)*	22 (42.3%)	0.026
**AG + GG**	37 (74%)	36 (72%)	25 (49%)^a^	30 (57.7%)	
**Genotype**					
**GG**	11 (22%)	8 (16.0%)	9 (17.6%)	6 (11.5%)	0.562
**AA + AG**	39 (40%)	42 (84%)	42 (82.4%)	46 (88.5%)	

The data are listed as frequencies (number of cases) and percentages.

*Significant increase compared to the control and HCV groups (*p* < 0.05).

^a^Significant decrease compared to the control and HCV groups (*p* < 0.05).

**Table 3:** The genotypes distribution of rs4143815 (C > G) polymorphism in the studied groups.

	**Control**	**HCV**	**HCV + Cirrhosis**	**HCV + HCC**	***P*****value**
	**(n = 50)**	**(n = 50)**	**(n = 51)**	**(n = 52)**	
**Genotype**					
**CG**	9 (18%)	8 (16.0%)	10 (19.6%)	20 (38.5%)	< 0.001
**CC**	21 (42%)	25 (50.0%)	1 (2%)	1 (1.9%)	
**GG**	20 (40%)	17 (34.0%)	40 (78.4%)	31 (59.6%)	
**Genotype**					
**CG**	9 (18%)	8 (16.0%)	10 (19.6%)	20 (38.5%)*^, ¥^	0.024
**CC + GG**	41 (82%)	42 (84.0%)	41 (80.4%)	32 (61.5%)^a, b^	
**Genotype**					
**CC**	21 (42%)	25 (50.0%)	1 (2%)^c^	1 (1.9%)^c^	< 0.001
**CG + CC**	29 (58%)	25 (50.0%)	50 (98%)**	51 (98.1%)**	
**Genotype**					
**GG**	20 (40%)	17 (34%)	40 (78.4%)**	31 (59.6%)*	< 0.001
**CG + CC**	30 (60%)	33 (66%)	11 (21.6%)^c^	21 (40.4%)^a^	

The data are listed as frequencies (number of cases) and percentages.

*Significant increase compared to the control and HCV groups (*p* < 0.05); ^¥^Significant increase compared to the cirrhosis group (*p* < 0.05); **Significant increase compared the control and HCV groups (*p* < 0.001); ^a^Significant decrease compared to the control and HCV groups (*p* < 0.05); ^b^Significant decrease compared to the cirrhosis group (*p* < 0.05); ^c^Significant decrease compared to the control and HCV groups (*p* < 0.001).

The distribution of rs4143815 (C > G) GG genotype was associated with a substantially increased risk of liver cirrhosis compared to the control and HCV groups (OR = 5.46, 95% CI = 2.27–13.08; *p* < 0.001 and OR = 7.06, 95% CI = 2.91–17.15; *p* < 0.001, respectively). The rs4143815 (C > G) CG and GG genotypes were associated with a considerably higher susceptibility to HCC development when compared to the control (OR = 2.85, 95% CI = 1.14–7.09, *p* = 0.02 and OR = 2.21, 95% CI = 1.00–4.89, *p* = 0.04, respectively) and HCV (OR = 3.28, 95% CI = 1.28–8.40, *p* = 0.01 and OR = 2.86, 95% CI = 1.28–6.41, *p* = 0.01, respectively) groups. Also, individuals carrying the CG genotype were at a higher risk of developing HCC, compared to the liver cirrhosis group (OR = 2.56, 95% CI = 1.05–6.23, *p* = 0.035) (Table 3).

Additionally, the distribution of rs4143815 (C > G) CG and GG genotypes was significantly positively associated with the clinicopathological features of HCC, including the Child-Pugh score (*p* < 0.001 and *p* = 0.002, respectively), BCLC stage (*p* < 0.001 and *p* = 0.011, respectively), and tumor lesion size (*p* = 0.007 and *p* = 0.007, respectively). Nevertheless, no association was observed between the CG and GG genotypes and the MELD/Na score (*p* = 0.112 and *p* = 0.214, respectively) (Table 4).

**Table 4:** The association of rs4143815 (C > G) CG and GG genotypes with the degree of the clinicopathological features of HCC.

	**CG**	***P*****value**	**GG**	***P*****value**
	**(n = 20)**		**(n = 31)**	
**Child-Pugh score**				
**A**	2 (10.0%)	< 0.001	3 (9.7%)	0.002
**B**	3 (15.0%)		9 (29.0%)	
**C**	15 (75.0%)		19 (61.3%)	
**MELD/Na score**				
**≤ 9**	2 (10.0%)	0.112	3 (9.7%)	0.214
**10**–**19**	6 (30.0%)		11 (35.5%)	
**20**–**29**	9 (45.0%)		9 (29.0%)	
**≥ 30**	3 (15.0%)		8 (25.8%)	
**BCLC stage**				
**A**	1 (5.0%)	< 0.001	0 (0.0%)	0.011
**B**	2 (10.0%)		5 (16.1%)	
**C**	3 (15.0%)		8 (25.8%)	
**D**	14 (70.0%)		18 (58.1%)	
**Lesion size**				
**< 3 cm**	4 (20.0%)	0.007	8 (25.8%)	0.007
**≥ 3 cm**	16 (80.0%)		23 (74.2%)	

On the other hand, the rs4143815 (C > G) CC genotype was found to be protective against the development of liver cirrhosis and HCC compared to the control (OR = 0.028, 95% CI = 0.004–0.22, *p* < 0.001 and OR = 0.027, 95% CI = 0.003–0.21, *p* < 0.001, respectively) and HCV (OR = 0.020, 95% CI = 0.003–0.156, *p* < 0.001 and OR = 0.020, 95% CI = 0.003–0.153, *p* < 0.001, respectively) groups (Table 3).

## 4 Discussion

To the best of our knowledge, this is so far the ﬁrst study that investigated the association between PD-L1 polymorphisms and the risk of liver cirrhosis and hepatocellular carcinoma (HCC) development in hepatitis C virus (HCV) Egyptian patients. In the current study, it was found that the frequencies of rs2297136 (A > G) AA genotype (51%) and rs4143815 (C > G) GG genotype (78.4%) were significantly higher in the liver cirrhosis group than the control (26% and 40%, respectively) and HCV (28% and 34%, respectively) groups indicating their potential as risk factors for the development of liver cirrhosis. A greater risk of HCC development was noticed in individuals carrying the rs4143815 (C > G) SNP CG and GG genotypes whereas the CC genotype of rs4143815 (C > G) was protective against the development of liver cirrhosis and HCC. In contrast, an insignificant association was observed between rs2297136 (A > G) SNP and HCC development. We further evaluated the relationship between PD-L1 polymorphisms and the clinicopathological aspects of HCC and it was noticed that the rs4143815 (C > G) SNP CG and GG genotypes had a significant positive association with the Child score, BCLC stage, and size of the tumor lesion.

Hepatocarcinogenesis, the gradual transition of nonmalignant liver cells into HCC, is a multi-step process defined by the cumulative accumulation of epigenetic and genetic alterations at the molecular and cellular levels. Although hepatitis B virus (HBV) and HCV infections are well-known risk factors for HCC, only a fraction of infected patients develop HCC during their lifetime implying that viral, host, and environmental factors all play a role in HCC susceptibility and occurrence [11,12]. One of these factors is the population genomic variation with several candidate gene analyses stating correlations between single nucleotide polymorphisms (SNPs) and the prevalence of HCC [13,14].

Immunotherapy of HCC appears to be a tougher road comparing to what is experienced with other tumors [15]. Therefore, a huge effort is being expended in the quest for predictive biomarkers as well as the development of innovative therapies to improve the outcomes of HCC patients [16].

Recently, it has been discovered that the expression of PD-1 and its ligand, PD-L1, was induced on the HCV-specific CD4^+^ and CD8^+^ T cells, resulting in T-cell exhaustion and dysfunction. Additionally, the HCV core protein was found to stimulate substantial PD-L1 up-regulation *in vitro* on primary human Kupffer cells as well as monocytes. These could be essential mechanisms for the viral immune escape and its persistence during chronic HCV infection [17,18].

Considering the immune evasion role of PD-L1 in cancer development, genomic variations of the PD-L1 gene could influence the anti-tumor immune response and thus, could be closely associated with greater cancer risks [4]. The 3′-untranslated region (3′-UTR) of PD-L1 mRNA is one of the most critical regulatory regions of PD-L1 gene expression because it is targeted by many miRNAs which mediate gene translational repression. So, structural variations in this region, including deletions, insertions, duplications, and translocations, may lead to aberrant PD-L1 expression and the development of various malignancies. Consequently, genetic variations in PD-L1 3′-UTR could be employed as valuable genetic markers to determine cancer susceptibility [5].

Whether PD-L1 gene polymorphisms are associated with a higher risk of cancer development has not been ascertained yet. However, several studies have shown that SNPs of the PD-L1 gene are implicated in the susceptibility to various types of cancers in different ethnic populations [5,19]. In the current study, rs2297136 (A > G) and rs4143815 (C > G) PD-L1 SNPs were investigated as prospective risk factors and predictive biomarkers for the development of liver cirrhosis and HCC in chronic HCV Egyptian patients.

rs2297136 is found in the 3′-UTR of PD-L1 gene and serves as a binding site for miRNA-324-5p, miRNA‐632, and miRNA-296-5p. An A to G change, at this location, has been shown to enhance the binding power between PD-L1 mRNA and its regulatory miRNAs resulting in a decline of PD-L1 gene transcription and protein expression [10,20]. The results of the current study indicated that rs2297136 (A > G) AA genotype was significantly associated with the development of liver cirrhosis but not with HCC development. This contrasted with Du et al. [21] study where rs2297136 polymorphisms in 3′-UTR of PD-L1 gene were associated with a greater risk of non‐small‐cell lung cancer (NSCLC), metastases development, tumor inﬁltration, and disease stage. Also, they revealed that the G-allele carriers (individuals with AG or GG genotype) were at a significantly higher risk of NSCLC, compared with those having A-allele homozygotes. Likewise, Wu et al. [20] illustrated that the subjects carrying the rs2297136 (A > G) GG genotype had lower levels of PD‐L1 protein expression and were positively correlated with a worse prognosis of gastric cancer (GC). Also, PD‐L1 rs2297136 AA + AG was associated with an up-regulated PD‐L1 protein expression and functioned as an independent predictor of better prognosis only in patients who didn't receive postoperative chemotherapy. This disparity could be attributed to the ethnic heterogeneity as well as the differences in environmental factors that may influence cancer predisposition.

The rs4143815C > G SNP is also located in the 3′-untranslated region (3′-UTR) of PD-L1 gene. It has been reported that a C to G mutation at the 3′-UTR of PD-L1 mRNA disrupts miRNA-570 binding, subsequently attenuates the miRNA-mediated mRNA degradation, and eventually leads to up-regulation of PD-L1 expression implying that the rs4143815C > G SNP may be functional [22]. Several studies have been performed to investigate the association of rs4143815C > G with the susceptibility to develop different types of diseases and cancers, however the results have been contradictory [23,24]. In the present study, the distribution of rs4143815 (C > G) GG genotype was significantly higher in patients with liver cirrhosis. In addition, individuals with the rs4143815 (C > G) SNP CG and GG genotypes were more likely to develop HCC, and the frequency of these genotypes was positively correlated with the clinicopathological aspects of HCC. Conversely, individuals carrying the CC genotype were protected against developing liver cirrhosis and HCC. Similar to our findings, type 1 diabetes mellitus (T1DM) patients homozygous for the rs4143815 GG genotype had higher levels of autoantibodies-positive incidence than the C-allele carriers whereas people carrying the C-allele of rs4143815 were less susceptible to T1DM in the Chinese population [24].

In line with the current study, it has been revealed that the rs4143815 GG genotype was associated with an elevated risk of gastric, bladder, and ovarian cancers [23,25]. Likewise, a previous study demonstrated that the rs4143185 GG genotype was associated with the risk and prognosis of HCC while the CC genotype was associated with better overall survival in the Chinese Han population [19]. However, this correlation was not detected in other studies performed on esophageal squamous cell carcinoma suggesting that the same polymorphism may have distinct effects on the carcinogenesis process of various malignancies [26].

Yeo and his colleagues reported that the PD‐L1 rs4143815 GG genotype may be beneficial for predicting poor prognosis in lung adenocarcinoma as patients with this genotype had lower overall survival and disease-free survival compared to patients with other genotypes of PD-L1 rs4143815 [27]. As well, prior studies stated that the PD‐L1 rs4143815C > G was significantly associated with a worse survival outcome and relapse with NSCLC and breast cancer [10,28,29]. Furthermore, Lee et al. [30] investigated whether the PD-L1 gene polymorphisms could predict the clinical outcome of patients with advanced NSCLC after paclitaxel-cisplatin chemotherapy and they found that the rs4143815 CG SNP was signiﬁcantly associated with a better response under additive model for the G-allele. The results helped to identify patients who might beneﬁt from chemotherapy and to prevent unnecessary drug side effects. They also assessed the association between the polymorphisms of PD-L1 and overall survival in patients with NSCLC and found that the rs4143815 CG SNP could not be used as a predictor for the NSCLC prognosis. Moreover, Nomizo et al. [31] indicated that the CC and CG genotypes and C-allele of PD-L1 rs4143815 were significantly associated with a better objective response rate and progression-free survival in NSCLC patients treated with nivolumab, and therefore, they suggested that the PD-L1 rs4143815 may be a biomarker for identifying patients for whom nivolumab might be particularly beneficial.

In the current case-control study, all the participants were ethnically homogeneous Egyptians, which may have avoided the ethnicity-driven bias. However, there were certain constraints. Firstly, only two SNPs were investigated in this study. Secondly, the sample size was relatively modest. Therefore, the relationship between other SNPs of PD-L1 and liver cancer should be investigated in further studies with a larger sample size.

In conclusion, the PD-L1 rs2297136 AA and rs4143815 GG genotypes are associated with an increased risk of liver cirrhosis while the PD-L1 rs4143815 CG and GG genotypes are positively correlated with the development and clinicopathological features of liver cancer. So, these SNPs may be utilized as a biomarker to predict the cancer risk. As a result, HCV patients carrying the PD-L1 rs4143815 G-allele should be followed up regularly to facilitate the early identification and treatment of HCC.

## Author contributions

M.H.: Conceptualization; Data curation; Formal analysis; Methodology; Writing - original draft; Writing - review & editing. M.S.A.: Data curation; Methodology; Writing - original draft. Z.A., G.A., and H.H.K.S.: Conceptualization; Formal analysis; Supervision; Writing - review & editing. M.E.: Data curation; Methodology. A.I.: Conceptualization; Formal analysis; Methodology; Writing - review & editing. All authors have revised and approved the manuscript.

## Declaration of competing interest

The authors declare that they have no known competing financial interests or personal relationships that could have appeared to influence the work reported in this paper.

## Data Availability

All data generated or analyzed during this study are included in this published article.
